# Predicting the Global Distribution of *Solenopsis geminata* (Hymenoptera: Formicidae) under Climate Change Using the MaxEnt Model

**DOI:** 10.3390/insects12030229

**Published:** 2021-03-08

**Authors:** Cheol Min Lee, Dae-Seong Lee, Tae-Sung Kwon, Mohammad Athar, Young-Seuk Park

**Affiliations:** 1 California Department of Food and Agriculture, 2800 Gateway Oaks Drive, Sacramento, CA 95833, USA; leecheolmin77@gmail.com (C.M.L.); athar.tariq@cdfa.ca.gov (M.A.); 2 Department of Biology, Kyung Hee University, Dongdaemun, Seoul 02447, Korea; dleotjd520@khu.ac.kr; 3 Alpha Insect Diversity Lab., Nowon, Seoul 01746, Korea; insectcom@naver.com

**Keywords:** invasive species, tropical fire ant (*Solenopsis geminata*), climate change impacts, prediction model, potential distribution, global warming

## Abstract

**Simple Summary:**

Climate change influences the distribution of species. The tropical fire ant *Solenopsis geminata* (Hymenoptera: Formicidae) is a serious invasive species that damages the native ecosystem. In this study, we evaluated the current and future distribution of *S. geminata* under climate change using the ecological niche model. The model results showed that the favorable habitat area of *S. geminata* will expand to higher latitudes on a global scale due to future global warming. Some countries located in America and East Asia, such as Brazil, China, South Korea, the USA, and Uruguay, can be threatened by *S. geminata* due to climate change.

**Abstract:**

The tropical fire ant *Solenopsis geminata* (Hymenoptera: Formicidae) is a serious invasive species that causes a decline in agricultural production, damages infrastructure, and harms human health. This study was aimed to develop a model using the maximum entropy (MaxEnt) algorithm to predict the current and future distribution of *S. geminata* on a global scale for effective monitoring and management. In total, 669 occurrence sites of *S. geminata* and six bioclimatic variables of current and future climate change scenarios for 2050 and 2100 were used for the modeling. The annual mean temperature, annual precipitation, and precipitation in the driest quarter were the key influential factors for determining the distribution of *S. geminata*. Although the potential global distribution area of *S. geminata* is predicted to decrease slightly under global warming, the distribution of favorable habitats is predicted to expand to high latitudes under climate scenarios. In addition, some countries in America and East Asia, such as Brazil, China, South Korea, the USA, and Uruguay, are predicted to be threatened by *S. geminata* invasion under future climate change. These findings can facilitate the proactive management of *S*. *geminata* through monitoring, surveillance, and quarantine measures.

## 1. Introduction

Invasive species have markedly influenced native species, communities, and ecosystems and caused extensive damage to human health and the economy around the world [1,2]. Moreover, invasive species are widely accepted as one of the leading threats to biodiversity and ecosystem services through predation, competition, and disease transmission [3]. Doherty et al. [4] reported that invasive species are related to 58% of modern species extinctions included on the Red List of Threatened Species by the International Union for Conservation of Nature.

Ants are a particularly conspicuous invasive species, with more than 200 species having spread outside their native range [5,6]. Among them, *Solenopsis geminata*, the tropical fire ant, is one of the worst invasive species, which causes damage to human health, plants, animals, and artificial equipment [7,8,9]. *S. geminata* is native to tropical and temperate regions of the American continent [10]; however, this ant has spread around the world, to Africa, south and southeast Asia, and Australia [9,11,12], through international trade and human activities. It is now unclear whether some populations are native or introduced [10]. Therefore, effective measures are required to prevent and control the dispersal of *S. geminata*.

Climate change affects the natural range, abundance, and dispersal of invasive species [13,14,15]. Species distribution is strongly influenced by various environmental factors [16,17]; therefore, changes in factors such as temperature, precipitation, and humidity due to climate change will further affect the distribution of invasive species [14,18]. However, there is relatively little information on invasive species management related to the potential impacts of climate change.

Ecological niche models (ENMs) are commonly used to predict the environmental suitability and distribution of species [19]. The ecological niche is a fundamental biological factor determining species distribution [20]. Various ENMs have exhibited good performance for diverse species; for example, generalized linear models have been used to evaluate habitat conditions for lynx restoration [21], machine learning methods such as random forest and support vector machine have been used to predict the occurrence of insects and plants [20,22], and a boosted regression tree has been used for the potential global distribution of red imported fire ant [23]. Furthermore, CLIMEX, which is a mechanistic niche model [24], has been used to predict the distribution of various species according to the organism’s physiological tolerance parameters [7,25]. Among the many available ENM methods, the maximum entropy (MaxEnt) algorithm is considered an excellent tool with high prediction performance that has been widely used for various species [18,26,27]. It has the advantage of avoiding potential errors when the physiological information of a species is uncertain [28].

This study aimed to identify the key environmental variables that correlate with the distribution of *S. geminata* on a global scale and predict the current and future potential distribution of *S. geminata* in response to climate change scenarios using the MaxEnt model.

## 2. Materials and Methods

### 2.1. Species Occurrence Data

**Figure 1:**
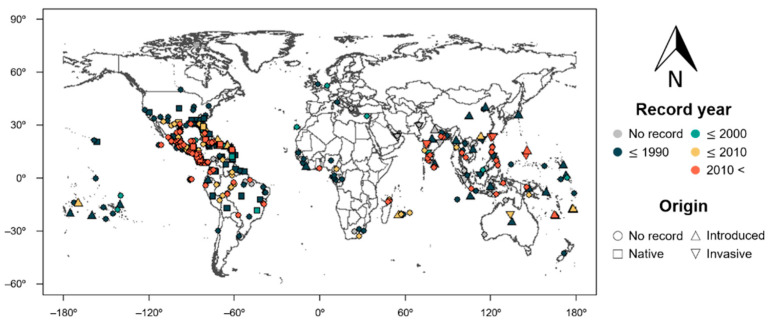
Global distribution of *Solenopsis geminata* based on databases of the Centre for Agriculture and Bioscience International and Global Biodiversity Information Facility. Different symbols and colors represent the different recorded years and origin of *S. geminata*, respectively, at each site. The data are available online [12,30].

Global distribution data of *S. geminata* were assembled from previous literature [9,29] and the databases of the Global Biodiversity Information Facility (GBIF) [12] and the Centre for Agriculture and Bioscience International (CABI) [30]. If only the localities were given, Google Earth (https://www.google.com/earth/) was used to collect the coordinates of the records. Records with obvious geocoding errors were discarded, and duplicate records were removed manually. Finally, 8194 sites of global *S. geminata* distribution were obtained for different periods (Figure 1).

### 2.2. Explanatory Variables

Bioclimate data of the CliMond climate dataset [31] were used as explanatory variables for both current and future climate conditions with a spatial resolution of 10 arc minutes. The CliMond climate data, consisting of 40 variables, have been widely used for predicting the current and future distributions of target species [7,25]. Among them, 19 core variables (Bio 1 to Bio 19) were used in this study. Some explanatory variables showing high collinearity between variables were excluded by using hierarchical cluster analysis with the complete linkage method based on the Spearman correlation distance [32]. From each cluster with relatively low correlation coefficients (<0.5), ecologically meaningful variables were selected empirically based on expert knowledge. This selection procedure led to a final set of six environmental variables: Annual mean temperature (Bio 1), maximum temperature of the warmest week (Bio 5), annual temperature range (Bio 7), annual precipitation (Bio 12), precipitation seasonality (Bio 15), and precipitation in the driest quarter (Bio 17).

The future climate of the CliMond dataset was based on a special report on emission scenarios (SRES) A1B and A2, developed by the CSIRO Mark 3.0 model [33]. The A1B scenario describes a balance between the use of fossil and non-fossil resources, whereas the A2 scenario describes a heterogeneous world with high population growth, slow economic development, and technological change [33]. This study used the future climate scenarios of SRES A1B and A2 for 2050 and 2100.

### 2.3. Modeling

The distribution of *S. geminata* was predicted by the MaxEnt model using the MaxEnt program (version 3.4.1) [26]. The MaxEnt model combines species presence data with randomly selected background data points from spatial environmental variables that represent different environmental gradients. The model then generates relative habitat suitability for a target species [26]. The MaxEnt model outperforms the majority of existing correlative modeling approaches and has been widely used to predict the potential distribution of insect pests [7,25,27].

Most species distribution models require spatially independent occurrence data for better performance. The spatially rarefy occurrence data tool in the SDMToolbox [34], which is a Python-based GIS toolkit for the spatial filtering of occurrence data to 1 km^2^, was used to reduce the sampling bias. Sites of indoor observations were excluded to ensure the reliability of the input data. Finally, the occurrence data were reduced to 669 sites using this procedure. Then, using the current and future bioclimate variables, the MaxEnt model predicted the climatic suitability of *S. geminata*.

The performance of the MaxEnt model is influenced by the choice of feature types and regularization constants in the model [35]. This feature presents a simple function of environmental variables and provides a set of constraints in MaxEnt modeling, whereas the regularization multiplier (constants) restricts excessive model complexity and mitigates model overfitting [26,35]. The MaxEnt program offers six features: Linear (L), quadratic (Q), product (P), threshold (T), hinge (H), and category indicator (C). Using the ENMeval package [36] in R software [37], the parameter setting was adjusted and 24 candidate models with different feature combinations (LQ, LQH, LQHP, and LQHPT) and regularization multipliers (1, 2, 5, 10, 15, and 20) were developed to select the best model for *S. geminata* distribution. The difference between the training and test area under the receiver operating characteristic curve (AUC_diff_) and Akaike’s information criterion (AIC_c_) were used to select optimal parameter combinations [19]. Through these processes, an optimal model was built with 5000 maximum iterations and 10,000 pseudo-absence points (i.e., model parameters).

### 2.4. Model Evaluation and Analysis

The performance of the MaxEnt model can be evaluated based on threshold-dependent and threshold-independent metrics. The MaxEnt model generates the AUC as a threshold-independent measure of model performance [26]. The higher the AUC value (closer to 1), the better the performance of the model, with good discrimination between the presence and absence of species [38]. The MaxEnt model was evaluated with a 10-fold cross-validation procedure. Averaged training and test AUC values were calculated across 10 replicates. The logistic output of the MaxEnt model represents the climatic suitability of the species on a scale from 0 to 1, with higher values representing more favorable conditions for the presence of the species [26]. In addition, a fixed cumulative value of 1 as a threshold-dependent metric was applied as a cutoff value to determine the suitable and unsuitable area of species distributions based on the omission rate. By contrast, other thresholds (such as the minimum training presence, 10 percentile training presence, and maximum test sensitivity plus specificity) had unacceptable omission rates or their predicted areas were believed to be ecologically inaccurate [26,39]. Finally, five classes of climatic suitability were determined as follows: Unsuitable (<0.0293), marginal (0.0293–0.2), moderate (0.2–0.4), favorable (0.4–0.6), and highly favorable (>0.6). The importance of each variable in predicting the species distribution was estimated by its contribution and permutation importance. Partial dependence plots were used to show the partial relationship (marginal effect) between each environmental variable and climatic suitability [40].

To compare the changes in distribution of *S. geminata* due to climate change, the surface area per class of climatic suitability was calculated, and the distribution of climatic suitability was analyzed by latitude and country. Local polynomial regression was used to fit the distribution of the habitat area of *S. geminata* according to latitude.

## 3. Results

**Figure 2:**
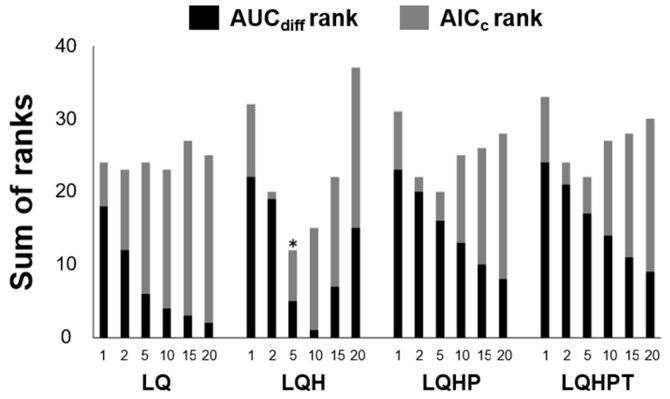
Selection of the best model based on the sum of AUC_diff_ and AIC_c_ ranks for 24 candidate models with different combinations of features (LQ, LQH, LHP, and LQHPT) and regularization multipliers (1, 2, 5, 10, 15, 20). Asterisk (*) represents the selected model (LQH5) showing the lowest sum of AUC_diff_ and AIC_c_ ranks.

*S. geminata* has a global distribution, but predominantly occurs in South and Central America, on the continents of Africa and Asia, and in the Pacific area (Figure 1). Based on records, the worldwide dispersal of *S. geminata* had already occurred prior to 1980. More recently, *S. geminata* has been observed in new areas such as Madagascar, Reunion, Cameroon, and Cambodia (Figure 1). The combination of feature types L, Q, and H and the regularization multiplier 5 (LQH5) was selected as the best model, as it exhibited the lowest sum of both AUC_diff_ and AIC_c_ ranks in the MaxEnt model (Figure 2). This model showed high levels of predictive performance with an AUC value of 0.923 ± 0.001 after model training and 0.907 ± 0.017 after model testing (average ± standard deviation).

**Figure 3:**
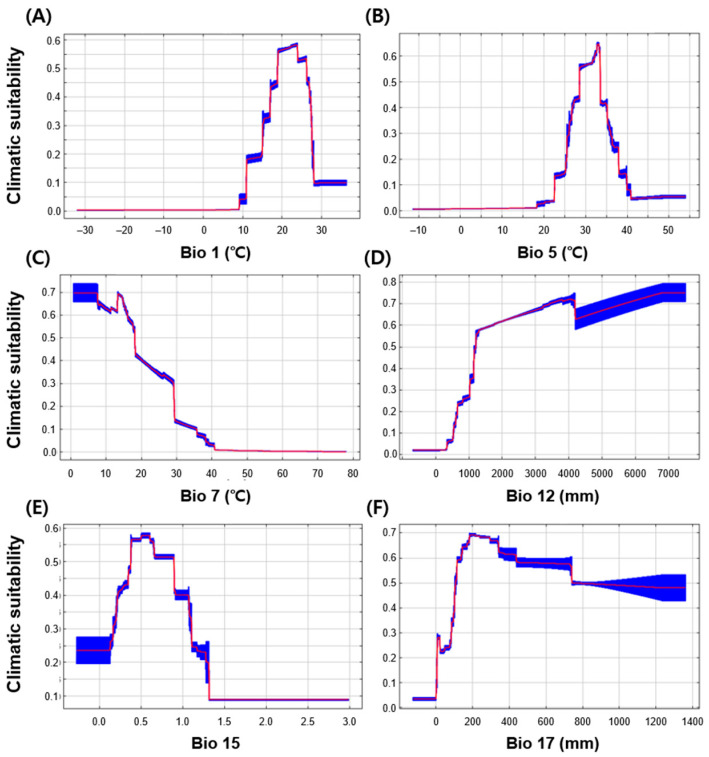
Response curves of MaxEnt model to changes in the six explanatory variables. Red lines and blue areas show the average and standard deviation of 10-fold cross-validation. (**A**) Bio 1: Annual mean temperature, (**B**) Bio 5: Maximum temperature of the warmest week, (**C**) Bio 7: Annual temperature range, (**D**) Bio 12: Annual precipitation, (**E**) Bio 15: Precipitation seasonality, (**F**) Bio 17: Precipitation in the driest quarter.

Among the explanatory variables, the most important variables showing high contributions and importance were the annual mean temperature (Bio 1), annual precipitation (Bio 12), and precipitation in the driest quarter (Bio 17, Table 1). Partial dependence plots revealed the response curves of six explanatory variables to the climatic suitability of *S. geminata* (Figure 3). The annual mean temperature range (Bio 1) was 9.3–36.3 °C, showing high response values at approximately 19–24 °C, and the maximum temperature of the warmest week (Bio 5) ranged from 18.4 to 54.0 °C, with a high response value at approximately 28–33 °C. The annual temperature range (Bio 7) had a negative impact on the occurrence of *S. geminata*, displaying a consistent decrease in its response when the annual temperature was increased, whereas the annual precipitation (Bio 12) had a positive impact on the occurrence of *S. geminata*. Furthermore, the precipitation seasonality (Bio 15) had the highest values at approximately 0.4–0.6. The peak precipitation range in the driest quarter (Bio 17) was approximately 197–238 mm, indicating a positive relationship with the occurrence of *S. geminata* for precipitation below 200 mm but a negative response for precipitation above 200 mm.

**Figure 4:**
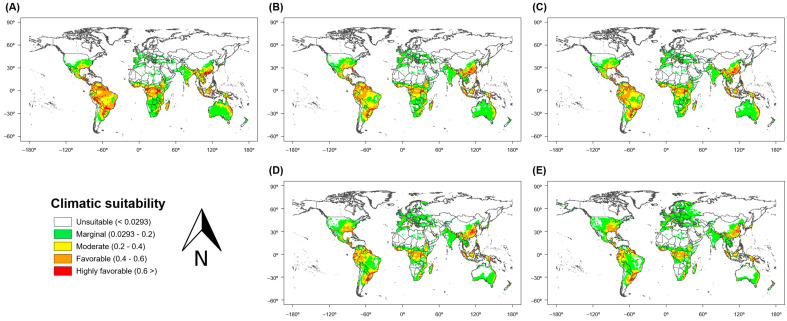
Climatic suitability for *S. geminata* on a global scale: (**A**) Under current climate conditions, (**B**) in 2050 under scenario A1B and (**C**) scenario A2, and (**D**) in 2100 under scenario A1B and (**E**) scenario A2.

The MaxEnt model predicted that the potential habitats of *S. geminata* will be widely distributed on a global scale (Figure 4). Under the current climate, 54.4% of the land area on Earth was deemed unsuitable (climatic suitability < 0.0293) for the distribution of *S. geminata*. The remaining area was divided into 16.9% of potentially marginal land (0.0293–0.2), 14.0% of potentially moderate land (0.2–0.4), 11.4% of potentially favorable land (0.4–0.6), and only 3.3% of potentially highly favorable land (>0.6) for *S. geminata*. The potentially favorable areas of *S. geminata* (>0.4) were predominantly located in some countries of Central and South America (USA, Brazil, and Colombia), Central Africa (Congo), South East Asia (Indonesia and China), and Australia. According to the MaxEnt model, global warming will induce a progressive change in the extent of suitable areas for *S. geminata* compared with the potential current distribution (Figure 4B–E). The suitable area of *S. geminata* (excluding unsuitable areas) was predicted to decrease slightly due to global warming. Under SRES scenarios A1B and A2, the proportion of suitable areas for *S. geminata* on Earth was predicted to change from 45.6% to 45.6% and 45.7% in 2050, and to 44.5% and 44.7% in 2100, respectively. In addition, the total favorable and highly favorable areas of *S. geminata* were also predicted to decrease due to global warming, from 14.7% to 11.9% and 12.1% in 2050 and from 14.7% to 9.6% and 8.2% in 2100 under SRES scenarios A1B and A2, respectively.

**Figure 5:**
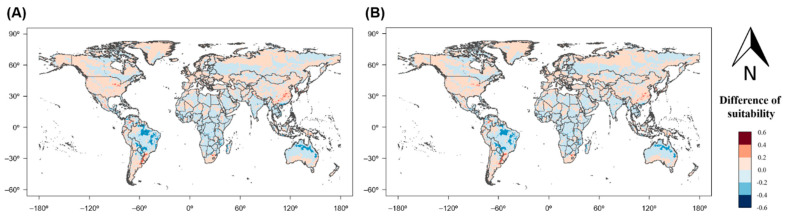
Changes in climatic suitability for *S. geminata* under global warming between current conditions and 2050 based on (**A**) scenario A1B and (**B**) scenario A2.

**Figure 6:**
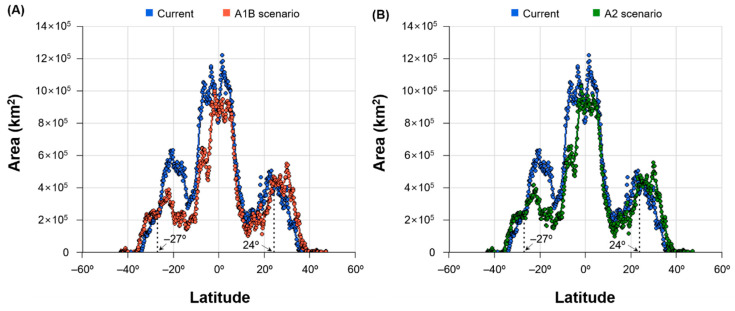
Distribution of favorable habitat areas of *S. geminata* (climatic suitability > 0.4) according to latitude under current climate and global warming scenarios (**A**) A1B and (**B**) A2 in 2050. Points indicate the total area per latitude and the lines represent smoothing performed by local polynomial regression. Vertical dotted lines show the boundary latitude at which the favorable habitat area of *S. geminata* is increased by global warming.

Predicted changes in climatic suitability due to global warming were large in Central and South America, East Asia, and Australia (Figure 5). Under SRES scenarios A1B and A2 for 2050, the climatic suitability of *S. geminata* was greatly decreased in some parts of Brazil and Australia. By contrast, it was greatly increased in other parts of Brazil, China, South Korea, and South Africa. In addition, the potential area of *S. geminata* was more widely distributed beyond the existing natural boundary under global warming (Figure 6). The future favorable habitat for *S. geminata* (climatic suitability > 0.4) was expanded to higher latitudes than the current favorable habitat. Above S27° and N24° latitude (to polar regions), the favorable habitat area for *S. geminata* increased from 2,491,249 km^2^ to 3,702,992 km^2^ and 3,604,058 km^2^ under SRES scenarios A1B and A2 in 2050, respectively.

**Figure 7:**
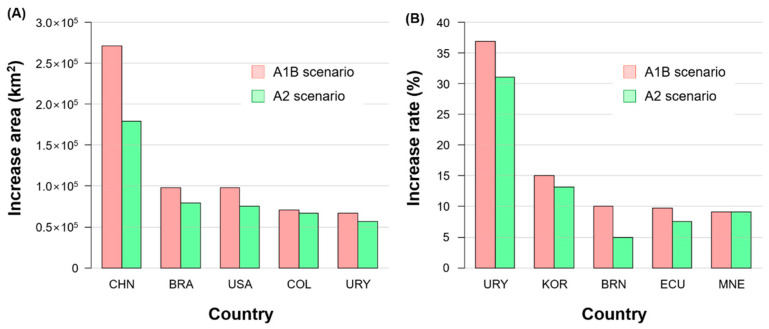
Top five countries for (**A**) increased area and (**B**) increased rate of climatic suitability (>0.2) for *S. geminata* in the future (2050) compared to current conditions according to global warming scenarios A1B and A2. BRA: Brazil, BRN: Brunei Darussalam, CHN: China, COL: Colombia, ECU: Ecuador, KOR: Republic of Korea, MNE: Montenegro, URY: Uruguay, and USA: United States of America.

Thus, global warming was predicted to expand the threat of *S. geminata* to several countries (Figure 7). The areas of increased climatic suitability for *S. geminata* (>0.2) due to global warming were predominantly in China, Brazil, the USA, Colombia, and Uruguay (Figure 7A). In addition, some countries such as Uruguay, South Korea, and Brunei Darussalam exhibited increased climatic suitability for *S. geminata* (>0.2) in more than 10% of their total areas under the SRES scenario A1B (Figure 7B).

## 4. Discussion

*S. geminata* already boasts a wide global distribution and is currently becoming more dispersed. Furthermore, it causes substantial ecological damage to invaded areas [9,41]. *S. geminata* is native to the area from south Texas in the USA to Central America and Brazil in northern South America [10,11]. However, it has since spread almost around the world to Europe, Africa, Asia, and Australia [7,8,9,11]. In particular, habitats of *S. geminata* have been observed all over Australia [42], although these sites were not updated in the GBIF and CABI databases. The distribution of *S. geminata* is influenced by various environmental factors, and climate factors are critical for determining its distribution on a large scale. Therefore, this study predicted the potential distribution of *S. geminata*, which can facilitate proactive management such as monitoring, surveillance, and quarantine measures.

The impacts of climate change on the geographical range and climatic suitability of areas for *S. geminata* were explored using the MaxEnt model in this study. Among the environmental variables used in the model, the annual mean temperature was most important as it strongly affected the distribution of *S. geminata*. The climate suitability for *S. geminata* responded positively to annual mean temperature in the temperature range of 19–24 °C and exhibited a negative relationship at temperatures higher than 25 °C. Annual precipitation and the range of precipitation in the driest quarter were also important variables in the model. Environmental factors, including temperature and humidity, affect the activity of ant colonies [43]. *S. geminata* is native to tropical areas, indicating that the species is adapted to warm climates, although it is predominantly active at night to avoid the highest ground temperature in the day [44,45]. Therefore, the behavioral activity of this species, i.e., avoiding high temperatures during the daytime, might be reflected in the results of the model.

The current potential global distribution of *S. geminata* predicted by the MaxEnt model indicated high performance of the model, as it included the current actual distribution areas for this species. For example, potential marginal and/or moderate areas were found in England, Italy, Portugal, Spain, France, and Eastern Europe, which is consistent with the known distribution of *S. geminata* in Italy, England, Greece, Cyprus, and the Netherlands. Although previous records of these countries mainly involved indoor observations [9], *S. geminata* can survive in the natural conditions of these areas. Furthermore, the current potential distribution predicted by the model involved some parts of North and Central Africa, Australia, and East Asia, where *S. geminata* has not yet been recorded. This indicates that the current potential distribution area might be broader than the actual observed area.

According to the MaxEnt model, habitat changes of *S. geminata* were more substantial under the high-concentration scenario (A2) in 2100 than under the low-concentration scenario (A1B). In some areas such as central Brazil, potentially favorable and highly favorable areas were predicted to decrease, suggesting that the natural range of *S. geminata* might decline under the impact of climate change. Meanwhile, the distribution of *S. geminata* was predicted to expand substantially to high latitudes under future global warming on the global scale, indicating that the distribution of *S. geminata* would be shifted to areas located at high latitudes. The MaxEnt model also predicted that many countries would be influenced by *S. geminata* in the future. Among them, countries located in America and East Asia, such as Brazil, Brunei Darussalam, China, Colombia, Ecuador, South Korea, Uruguay, and the USA, were considered high-risk areas of *S. geminata* invasion.

The spread of *S. geminata* is increasing due to anthropogenic causes. Among the countries at risk of *S. geminata* invasion predicted in this study, *S. geminata* has not been introduced to Montenegro or South Korea. However, in South Korea, reports of *S. geminata* detection during plant quarantine have been steadily increasing [46] from one case in 1990 to seven cases in 2006; it is now listed as a regulated pest in the Plant Quarantine Act of Korea. In addition to the predicted at-risk countries, many other countries are also in danger of *S. geminata* invasion. The MaxEnt model predicted that *S. geminata* can live in almost all countries except microthermal and arid climate zones. Therefore, the results of the MaxEnt model show that these countries should establish an intensive plant quarantine program and control strategy to protect their ecosystems from the spread of *S. geminata*.

ENMs have previously supported the development of strategies for invasive species and ecosystem management [47,48]. In addition, ecological modeling plays an important role in eradication programs of invasive species, particularly when limited resources are available [48,49]. However, there have been limited studies on *S. geminata* using modeling approaches. For example, Baker et al. [49] developed two models (a population model and a detection model) to propose an efficient framework for *S. geminata* control projects on the islands of Ashmore Reef in the Timor Sea. Byeon et al. [7] used a CLIMEX model to predict the potential distribution of *S. geminata* according to climatic suitability on a global scale. Our MaxEnt model results predicted a smaller potential distribution of suitable areas under current and global warming scenarios for 2050 and 2100 than the CLIMEX model by Byeon et al. [7]. In particular, there was a large difference between the percentage of favorable areas for *S. geminata* in our study (area of climatic suitability > 0.4) and in the CLIMEX model (area of ecoclimatic index ≥30 in Byeon et al. [7]) under all climate conditions. This might be a result of the properties of each model. The MaxEnt model is closer to the realized niche than the fundamental (physiological) niche because the model was not built using the physiological traits of *S. geminata* [50]. In addition, the MaxEnt model showed more conservative results than the CLIMEX model in the prediction of an agricultural insect pest with global climatic suitability [25]. Furthermore, the study of Byeon et al. [7] lacked information related to the influence of environmental variables on the prediction of *S. geminata*, whereas our model showed that the annual mean temperature, annual precipitation, and precipitation in the driest quarter were the key factors influencing the distribution of *S. geminata*.

The dispersal and distribution of species are affected by physical environmental factors, as well as biological factors [16,17]. In this study, the effects of climatic factors were explored, which affect the biology and ecology of the species. Among them, the annual mean temperature, annual precipitation, and precipitation in the driest quarter were most influential on the global distribution of *S. geminata*. These factors affect the shift in species distribution under natural conditions through active movement of the species themselves, resulting in a short-range dispersal and low dispersal speed. The dispersal speed is positively dependent on the population density of the species [51]. However, many invasive species display a high dispersal speed and long-distance dispersal, which are highly related to human activities [51,52,53], indicating that human-mediated dispersal accelerates the dispersal speed of invasive species, such as the emerald ash borer (*Agrilus planipennis*) in the USA [54] and the citrus flatid planthopper (*Metcalfa pruinosa*) in Korea [22]. In particular, an increase in international trade and travel accelerates the dispersal of various invasive species on a global scale [55,56]. Meanwhile, human factors such as nighttime light and urban accessibility can make considerable contributions to the dispersal of species [23], and habitat disturbance caused by anthropogenic, as well as natural, factors can trigger the local expansion of species [57].

In this study, the model was not developed to predict the dispersal of *S. geminata* but to predict its potential distribution in the future considering bioclimatic factors. These model results may also be influenced by other variables, such as physical habitat conditions and the adaptation ability of invasive species [56,58], which were not included in this study. In general, invasive species easily adapt to new environmental conditions and rapidly spread to new regions [59,60]. Therefore, further studies are required to evaluate the distribution of species considering the influence of biological adaptation, as well as physical habitat conditions. A dispersal model of invasive species is also required to predict the dispersal patterns and distribution areas over time on both global and regional scales.

## 5. Conclusions

In this study, we predicted the potential distribution of *S. geminata* on a global scale using the MaxEnt model, which was based on bioclimatic factors. The model exhibited high prediction performance. The annual mean temperature, annual precipitation, and precipitation in the driest quarter were the key factors influencing the potential distribution of *S. geminata*. Although the potential distribution areas of *S. geminata* were predicted to decrease slightly on a global scale due to future global warming, the distribution of favorable habitats for *S. geminata* was predicted to expand and shift to high latitudes. In addition, some countries located in America and East Asia, such as Brazil, China, South Korea, the USA, and Uruguay, were considered high-risk areas of *S. geminata* invasion. The results of this study provide baseline data to facilitate the proactive management of *S. geminata* through monitoring, surveillance, and quarantine measures.

## Figures and Tables

**Table 1:** Contribution (%) and permutation importance of environmental variables in predicting the occurrence of *S. geminata* in the MaxEnt model.

Variable	Contribution (%)	Permutation Importance
Bio 1	44.5	60.5
Bio 5	0.7	1.5
Bio 7	7.0	1.6
Bio 12	28.2	2.4
Bio 15	2.8	6.0
Bio 17	17.0	28.1

## Data Availability

The data presented in this study are available in this article.
